# Material priority engineered metal-polyphenol networks: mechanism and platform for multifunctionalities

**DOI:** 10.1186/s12951-022-01438-1

**Published:** 2022-06-03

**Authors:** Xinxiu Cheng, Yaxin Zhu, Sicheng Tang, Ruofei Lu, Xiaoqiang Zhang, Na Li, Xingjie Zan

**Affiliations:** 1 Oujiang Laboratory (Zhejiang Lab for Rengerative Medicine, Vision and Brain Health), Wenzhou Institute, University of Chinese Academy of Sciences, Jinlian Rd. 1, Wenzhou, 325001 People’s Republic of China; 2 Xinjiang Technical Institute of Physics and Chemistry, Chinese Academy of Sciences, Urumqi, 830011 People’s Republic of China

**Keywords:** Metal-polyphenol networks, Procyanidin, Tissue engineering, Multifunctionalities, Mechanism

## Abstract

Engineering the surface of materials with desired multifunctionalities is an effective way to fight against multiple adverse factors during tissue repair process. Recently, metal-polyphenol networks (MPNs) have gained increasing attention because of their rapid and simple deposition process onto various substrates (silicon, quartz, gold and polypropylene sheets, etc.). However, the coating mechanism has not been clarified, and multifunctionalized MPNs remain unexplored. Herein, the flavonoid polyphenol procyanidin (PC) was selected to form PC-MPN coatings with Fe^3+^, and the effects of different assembly parameters, including pH, molar ratio between PC and Fe^3+^, and material priority during coating formation, were thoroughly evaluated. We found that the material priority (addition sequence of PC and Fe^3+^) had a great influence on the thickness of the formed PC-MPNs. Various surface techniques (e.g., ultraviolet–visible spectrophotometry, quartz crystal microbalance, X-ray photoelectron spectroscopy, atomic force microscopy, and scanning electron microscopy) were used to investigate the formation mechanism of PC-MPNs. Then PC-MPNs were further engineered with multifunctionalities (fastening cellular attachment in the early stage, promoting long-term cellular proliferation, antioxidation and antibacterial activity). We believe that these findings could further reveal the coating formation mechanism of MPNs and guide the future design of MPN coatings with multifunctionalities, thereby greatly broadening their application prospects, such as in sensors, environments, drug delivery, and tissue engineering.

**Graphical Abstract:**

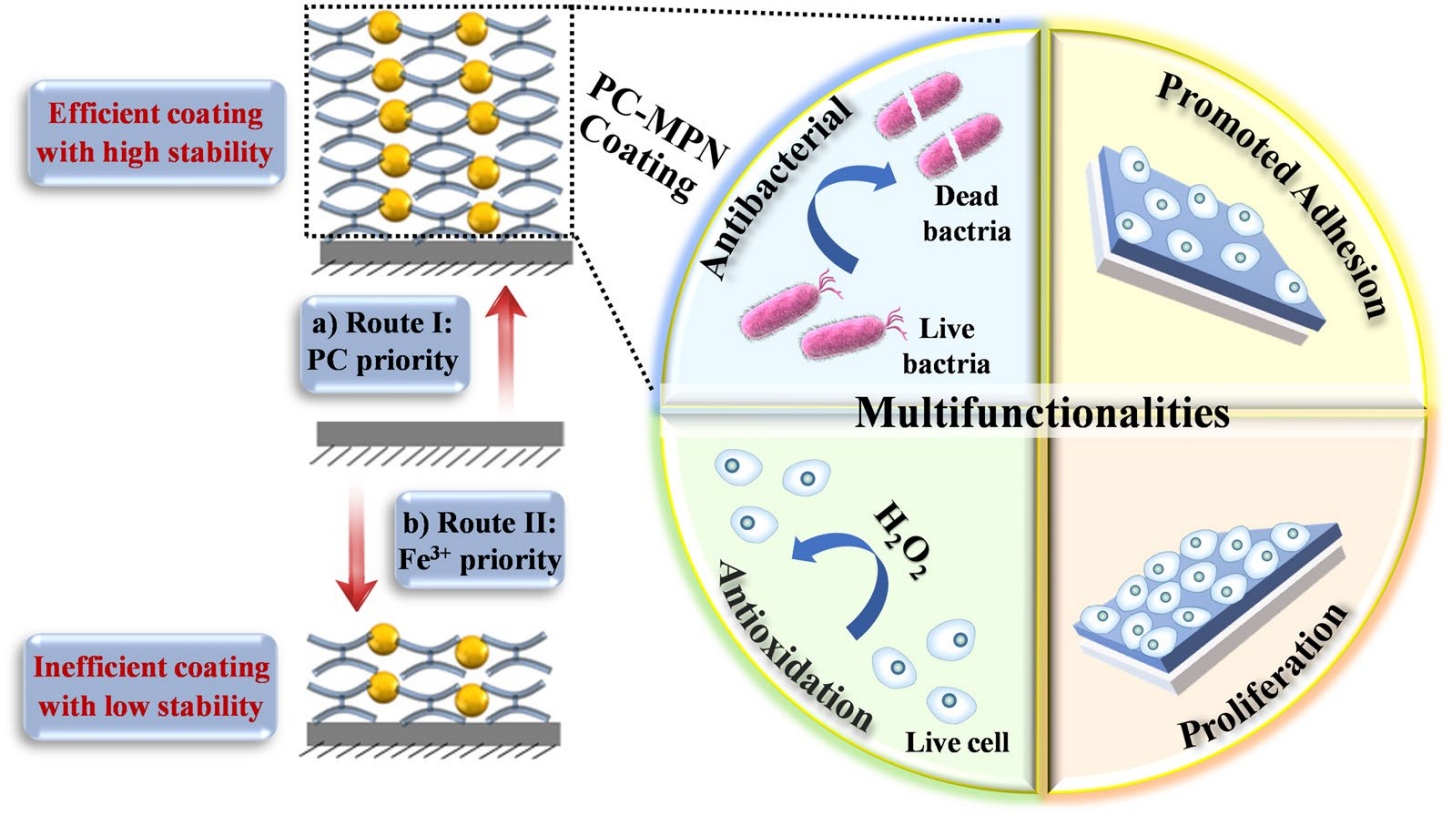

**Supplementary Information:**

The online version contains supplementary material available at 10.1186/s12951-022-01438-1.

## Introduction

Surface is the first point of interaction when two materials are in contact with each other, whose functionalities are crucial to the performance of the material [1–4]. Implantation is a complicated process that is orchestrated by various cellular and biomolecular signals in a spatiotemporally defined manner to achieve expected outcomes [5, 6]. When implantation happens, a series of biological events could occur at the interface between implants and surrounding tissues, such as protein adsorption, cell attachment, infection, inflammation, or coagulation [7–10]. Thus, engineering the surface of implants with desired multifunctionalities has been proposed as an effective way to fight against multiple adverse factors during the tissue repair process [11–14]. For example, orthopedic implants equipped with antibacterial properties enhanced tissue integration, improved bone regeneration, and could reduce the current high failure rate [7] (approximately 20% after 10 years of implantation based on multicenter data), which may be caused by multiple factors, such as infection, poor biocompatibility, and slow or incomplete bone regeneration [10]. However, developing a multifunctional coating based on a simple and universal method remains a significant challenge.

Because of their rapid and simple deposition process onto various substrates with different shapes, metal-polyphenol networks (MPNs), constructed from the dynamic coordination between metal ions and phenolic ligands, have gained increasing attention since they were first reported by Caruso et al. [15]. Recently, pioneering explorations of multifunctional MPN coatings have been performed due to their ability to combine the functions of polyphenol and metal ions. It allows the functionalities of the formed coating to be endowed or synergistically promoted by the applied polyphenol and metal ions. Huang et al. explored the MPNs formed by polyphenol (tannic acid)/catecholamine (dopamine or norepinephrine) and copper (II) ions for a potential coating on blood-contacting implantable devices because of its synergistic anti-inflammatory, antimicrobial and anticoagulant properties [16]. Taking advantage of the coating formed by epigallocatechin gallate (EGCG) and magnesium ions, Wang and colleagues [17] demonstrated its ability to enhance corrosive resistance and endothelialization for potential cardiovascular applications. Shin’s group [18] showed that the EGCG/Mg^2+^ coating had a promising ability to promote osseointegration and bone formation and thus represented a reliable surface modification on orthopedic implants. Stimulated by the broad applications, the mechanism of MPNs formation gains a lot of attention and has been extensively investigated. It has been reported that the assembly process could be influenced by many factors, such as the pH [15], solvents [19], precursor concentration [19], molar ratio between polyphenols and metal ions [15], and even the addition sequence of polyphenols and metal ions [20, 21]. However, in most current reports, the formation of MPNs is based on tannic acid (TA) [22], which is a tip of the iceberg considering the large families of polyphenols (more than 8000 species of polyphenols have been identified) [23, 24]. In a recent report, Caruso et al. demonstrated that a small change in the number of possible chelating sites of polyphenols with metal ions resulted in a profound formation of MPNs [25]. Considering the large number of polyphenols and the wide range of appliable metal ions (Fe^3+^, Cu^2+^, Mg^2+^, etc.) [26], it is reasonable to envision that the functionality world of MPNs must be developed urgently, and the formation mechanism of MPNs is far from being fully understood.

**Scheme 1:**
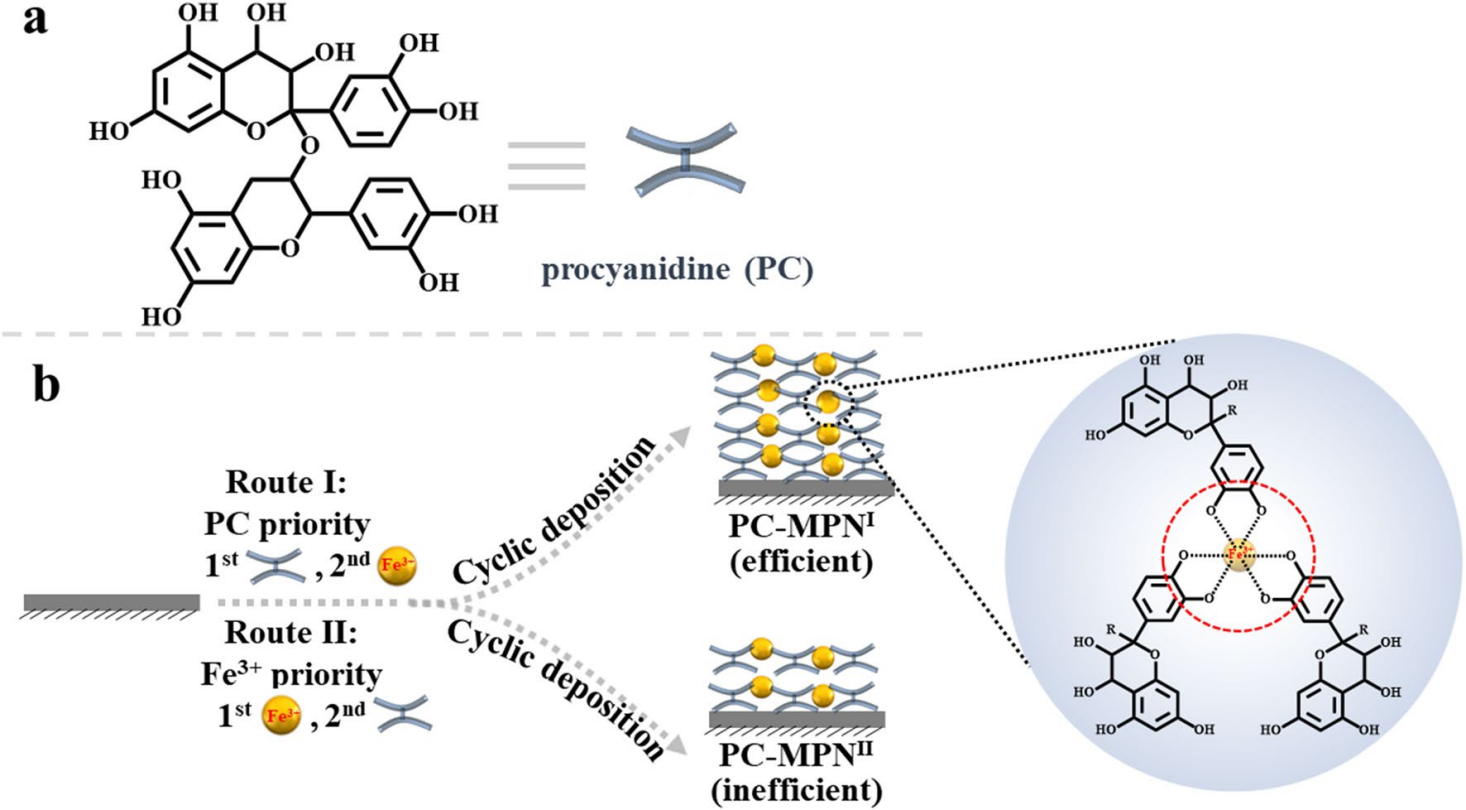
**a** Chemical structure of procyanidin (PC). **b** Schematic illustration of PC-MPN^I^ and PC-MPN^II^ coating production by route I and route II, respectively

Polyphenols, based on their carbon backbone structure, can be classified into four categories: phenolic acids, flavonoids, stilbenes and lignans, among which phenolic acids and flavonoids are the most common ones [23, 27, 28]. Compared with TA (a representative phenolic acid polyphenol with a C6-C1 structure) [24], which is a representative polyphenol that is typically used to study the formation mechanism of MPNs, procyanidin (PC, a homolog to flavonoid polyphenol, with a C6-C3-C6 structure, Scheme 1a) [24, 29], has rarely been investigated. PC is widely present in nature, especially in seeds, fruits, flowers, bark, nuts and vegetables, and exhibits excellent bioactivities, such as antioxidant activity [30], antibacterial activity [31], insect pest resistance [32], antiobesity activity [33], antithrombosis activity [34], bone osteogenesis promotion activity [35] and platelet activity modulation ability [36]. Herein, PC and iron ions (Fe^3+^, a classic metal ion of MPNs) were chosen to investigate PC’s ability to construct MPNs (PC-MPNs) by varying the factors affecting MPN formation and the multifunctionality of PC-MPNs. We surprisingly found that the thickness of PC-MPNs was greatly affected by pH, molar ratio of PC to metal ions (Fe^3+^) and the sequence in which PC and Fe^3+^ were added. With a focus on the addition sequence of PC and Fe^3+^, we systematically explored the formation mechanism of PC-MPNs and further engineered PC-MPNs for specific multifunctionalities, including cellular attachment and proliferation accelerating strong antioxidant ability, and antibacterial capacity. We believe that our findings on the PC-MPN coating formation mechanism suggest that the strategy of engineering a PC-MPN coating multifunctional platform has considerable application prospects and could further guide the future design of other MPN coatings.

## Materials and methods

### Materials

Proanthocyanidin (PC), iron(III) nitrate nonahydrate (Fe(NO_3_)_3_∙9H_2_O), N-2-hydroxyethyl piperazine-N'-2-sodium sulfonate salt (HEPES-Na), bis-(2-hydroxyethyl)amino-tris(hydroxymethyl)methane (bis–tris), silver nitrate (Ag(NO_3_)_3_) and tris(hydroxymethyl)aminomethane (tris) were purchased from Macklin. NH_3_∙H_2_O, H_2_SO_4_, H_2_O_2_ (30%), phosphate-buffered saline (PBS), 4′,6-diamidino-2-phenylindole (DAPI), rhodamine phalloidin (phalloidin-TRITC) and polypropylene (PP) sheets were purchased from Sigma. Four percent paraformaldehyde and Triton X-100 were purchased from Solarbio Life Science. Cell Counting Kit-8 (CCK-8) was obtained from Dojindo Laboratories. The ROS assay kit and ferric reducing antioxidant power (FRAP) assay kit were purchased from Beyotime Institute of Biotechnology. All chemicals were used without any further purification. Silicon wafers (SSPs), quartz plates (Alfa Aesar), and round glass coverslips (14 mm diameter from NEST) were cleaned by piranha solution (70% H_2_SO_4_ and 30% H_2_O_2_, V/V) at 98 ℃ for 2 h, rinsed with Milli-Q water and subsequently dried under a mild stream of air before use (Caution: Piranha solution is highly oxidizing and corrosive, and extreme care should be taken during preparation and use). The deionized (DI) water used here was purified through a Milli-Q system and possessed a resistivity greater than 18.25 MΩ·cm.

### Preparation of PC-MPN

PC was dissolved in water at a concentration of 1 mM, and NaOH (2 M) was dropped into the above PC solution to adjust the pH to 8. Equal volumes of mixed buffer (pH 6.5) containing bis–tris (0.3 M) and tris (0.3 M) were added to the PC solution (1 mM, pH 8). The resulting solution was evenly mixed and subsequently centrifuged at 8600 rpm for 7 min to remove undissolved PC, and it was denoted as the stock solution. Fe(NO_3_)_3_ solution (5 mM) was obtained by dissolving Fe(NO_3_)_3_ in water. PC-MPNs could be coated through two routes (depending on the priority of the first added material), as illustrated in Scheme 1b. PC-MPN coatings engineered by route I (PC priority) and route II (Fe^3+^ priority) were denoted PC-MPN^I^ and PC-MPN^II^, respectively. Fabricating PC-MPN^I^ coating was carried out in a 10 mL tube containing a substrate (clean silicon, quartz or PP sheet with 1 cm × 1 cm), in which 1.4 mL of stock solution was added, and then 0.7 mL of Fe(NO_3_)_3_ solution was added immediately (less than 10 s). The mixture was vortexed vigorously for 30 s, and the substrate was washed with HEPES buffer solution (0.025 M, ca. pH 8) and DI water. The substrate was then dried by a mild stream of air. The above deposition procedure of PC-MPNs was cycled until the desired thickness was obtained. The PC-MPN^II^ coating was produced by the same protocol, except that Fe(NO_3_)_3_ solution was first added followed by the addition of stock solution.

### Factors of PC-MPN fabrication

The effects of the pH and molar ratio of PC to Fe^3+^ (PC:Fe^3+^) PC-MPN fabrication were evaluated on a PC-MPN^I^ coating. For the pH factor, HCl or NaOH was used to adjust the pH values of the stock solution to the setup point of 6, 7 and 8. It should be noted that the pH had no variation during the whole coating process, regardless of the addition of the Fe(NO_3_)_3_ solution due to the effect of buffer in the stock solution. For the PC:Fe^3+^ factor, different ratios (1:2–1:10) were achieved by fixing the PC concentration with varying concentrations of Fe(NO_3_)_3_ solution. PC-MPN^I^ coatings at different pH values or different PC:Fe^3+^ ratios were obtained by the above protocol.

### Deposition process of PC-MPN^I^ and PC-MPN^II^ coatings

The deposition process of the PC-MPN^I^ and PC-MPN^II^ coatings was recorded by ultraviolet–visible (UV–vis) spectra. PC-MPN^I^ and PC-MPN^II^ coatings were coated onto quartz slides using a modified protocol, with the quartz slide washed and dried after each addition of stock solution and Fe(NO_3_)_3_ solution. The absorbance at 200–800 nm was recorded for further analysis. The time of quartz slide immersion into mother liquid or Fe(NO_3_)_3_ solution was set at 10 s.

### Decomposition process of PC-MPNs

Generally, the decomposition process of PC-MPNs was carried out by immersing a substrate coated with PC-MPNs into an Fe(NO_3_)_3_ solution (pH 2.6) or dilute HCl solution (pH 2.6) for the desired time (5 min and 30 min). Then the sample was then washed with DI water and dried under vacuum. The absorbance at 200–800 nm and the thickness and morphology of these coatings were recorded by UV–vis, ellipsometry and atomic force microscopy (AFM), respectively. The thickness was approximately 14 nm for both PC-MPN^I^ and PC-MPN^II^ coatings, and the MPNs were fabricated by 4 cyclic depositions for the PC-MPN^I^ coating and 20 cyclic depositions for the PC-MPN^II^ coating.

### Quartz crystal microbalance with dissipation (QCM-D) test

The QCM-D test was performed using a Q-sense system (Biolin Scientific, Sweden) with an E4 channel to further investigate the decomposition process of the coating. The gold-coated quartz crystals for QCM measurements were cleaned in an ammonium peroxide mixture (5:1:1, water: NH_3_(25%): H_2_O_2_ (30%)) at 75℃ for 0.5 h, rinsed with Milli-Q water, and dried with a mild stream of air [37]. The shifts in energy dissipation (ΔD) and resonance frequency (ΔF) were monitored in real-time under third overtones. For the study of the coating behavior in PC solution, the coating was first prepared on QCM-D resonators with seven deposition cycles. Then, cells were incubated in PC solution for 40 min. Following PC adsorption, rinsing solutions of bis–tris buffer and Milli-Q water were injected for 10 min each. A flow rate of 50 μL per min was maintained for every step. The study of the coating behavior in Fe(NO_3_)_3_ and HCl solutions was performed using the same protocol, except that cells were incubated in Fe(NO_3_)_3_ and HCl solutions for 40 min to allow adsorption. Next, rinsing solutions of Milli-Q water were injected for 10 min.

### FRAP text

The working curve was obtained first based on the protocol from the FRAP assay kit (Additional file 1: Figure S1). In brief, 27.8 mg FeSO_4_ solutions with different specific concentrations (0, 0.15, 0.3, 0.6, 0.9, 1.2 and 1.5 mM) were prepared and injected into a 96-well plate, into which working solution (180 µL) was added. After incubating for 5 min at 37℃, the 96-well plate was read at 593 nm with a spectrophotometer. The total antioxidant activities of the PC-MPN^I^ coatings, glass and troliox (1 mM) were measured with the same procedure, except that the PC-MPN^I^ coatings, glass and trolox were used instead of the FeSO_4_ solution for reacting with the FRAP working solution. Glass and trolox were run as the negative and positive control, respectively. After reacting for 5 min at 37℃, the samples were taken out, and the solution was read by a spectrophotometer at 593 nm. Based on the tested intensity, the antioxidant activity of each sample was calibrated from the standard curve. After 3 repetitions, the average data were reported.

### Cell culture

MC3T3-E1 cells (ATCC, subclone 14) were cultured and passaged onto a 75 mm^2^ tissue culture flask at 37℃ in humidified air containing 5% CO_2_ supplying with Dulbecco’s modified Eagle’s medium (DMEM) supplemented 10% fetal bovine serum, penicillin 100 (units/mL) and streptomycin (100 μg/mL).

### Cell attachment and spreading at early stage

MC3T3-E1 cells were collected and redispersed in culture medium. After counting the number of cells, MC3T3-E1 cells were seeded on glass coverslips with or without PC-MPN^I^ coatings at a density of 4 × 10^4^ cells/cm^2^. At 2 h and 4 h after seeding, the glass coverslips were taken out and washed softly with PBS, and then the cells were fixed by immersion in 4% paraformaldehyde for 30 min. The coverslips were further treated with PBS containing 0.2% (v/v) Triton X-100 for 10 min, followed by rinsing with PBS five times. Then, all coverslips were stained subsequently with actin filaments with the fluorescent dye phalloidin-TRITC (red), and nuclei were stained with DAPI (blue). The cell morphology was imaged by fluorescence microscopy (DMi8, Leica).

### Cell proliferation

Cell proliferation was tested on PC-MPN^I^ coatings by seeding MC3T3-E1 cells at a density of 1 × 10^4^ cells/cm^2^ in 24-well plates, with glass used as the control. After culturing for several days (1, 3 and 7 days), a CCK-8 kit was used to test the viability of cells with the procedure from the kit. Briefly, CCK-8 solution (10% of the total medium volume) was added to MC3T3-E1 cells. After 2 h of incubation, 100 μL aliquots from each sample were transferred to a 96-well plate and read at 450 nm. The data are reported as the average value from triplicates. After culturing for 7 days, the cells were fixed and stained by the previous procedure and imaged by fluorescence microscopy.

### Intracellular ROS level

MC3T3-E1 cells were seeded on coated glass coverslips at a density of 4 × 10^4^ cells/cm^2^. After 24 h of culture, the medium was replaced by DMEM with/without 10 mM H_2_O_2_. After 2 h of culture, the cells were washed gently with PBS 3 times. Cells were stained by the previous procedure and imaged by fluorescence microscopy.

### PC-MPN-Ag coating

PC-MPN^I^-Ag coating was generated by immersing silicon wafer or glass coated with PC-MPN^I^, which was fabricated by the protocol in the above “Preparation of PC-MPN section” with the optimized molar ratio (PC:Fe^3+^, 1:10) and pH 6.5, in Ag(NO_3_)_3_ solution (10 mM) for 24 h.

### Antibacterial test

Bacteria (*S. aureus* (ATCC #6538) and *E. coli* (ATCC #8739)) in lysogeny broth (LB) medium were harvested by centrifugation and resuspended in PBS buffer solutions (pH 7.2) to a final concentration of 1 × 10^8^ in 10 mL solution. The samples (PC-MPN^I^ and PC-MPN^I^-Ag coatings on silicon wafer or glass) and the control (silicon wafer or glass) were immersed in 1 mL of the above bacterial solution in 24-well plates and incubated for another 6 h at 37℃. Then, the samples and the control were washed three times with PBS. For the silicon wafer group, the sample substrates were immersed in paraformaldehyde (4%) for 15 min for prefixation and subsequently washed three times with PBS. After the samples and the control were dried under vacuum, field scanning electron microscopy (SEM) measurements were carried out to observe the morphological change in the bacterial cells. A LIVE/DEAD BacLight kit was used to further test the antibacterial ability of samples, where live and dead bacteria were stained green and red, respectively. The procedure was followed using the protocol provided by the kit. Finally, the morphological changes in the bacterial cells were imaged by confocal laser scanning microscopy (CLSM, A1 Nikon).

### Characterization

All PC-MPN^I^ and PC-MPN^II^ coatings were fabricated by strictly following the above “Preparation of PC-MPN section” with the optimized molar ratio (PC:Fe^3+^, 1:10) and pH 6.5. All as-prepared PC-MPN coatings were stored in a 4℃ refrigerator and fully dried before any characterization. The thickness was determined by optical ellipsometry (M-2000UI, J.A. Woollam). The surface morphologies were observed by AFM (Dimension Icon, Bruker) in tapping mode and SEM (SU8010, HITACHI) in SE (L) mode. UV–vis spectra of PC-MPN^I^ and PC-MPN^II^ coatings deposited on quartz slides were collected on a UV–vis spectrophotometer (Lambda 25, PerkinElmer). X-ray photoelectron spectroscopy (XPS) spectra of PC-MPN^I^ and PC-MPN^II^ coated on silicon wafers were obtained on a Thermo-Electron ESCALAB 250 spectrometer equipped with a monochromatic Al X-ray source (1486.6 eV). Water contact angle (WCA) analyses of PC-MPN^I^ and PC-MPN^II^ coated on PP sheets were carried out using the static sessile drop method on a KRUSS DSA1 version 1.80 drop shape analyzer, with water as the probe liquid. Each contact angle value represented the average of at least seven measurements.

### Data processing and statistical analysis

For all data, the average of at least three duplicates was reported, and the error bar indicated the standard deviation. For analysis of cell number and cell area, at least 10 images were randomly selected and calculated with the assistance of ImageJ, which was downloaded from its official website (https://imagej.net/downloads). The significant difference between each two interested groups was performed using the t-test, and was indicated by these symbols “*”, “**”, “***” and “****” for p < 0.05, p < 0.01, p < 0.001 and p < 0.0001, respectively. No significance was noted as “ns”.

## Results and discussion

**Fig. 1:**
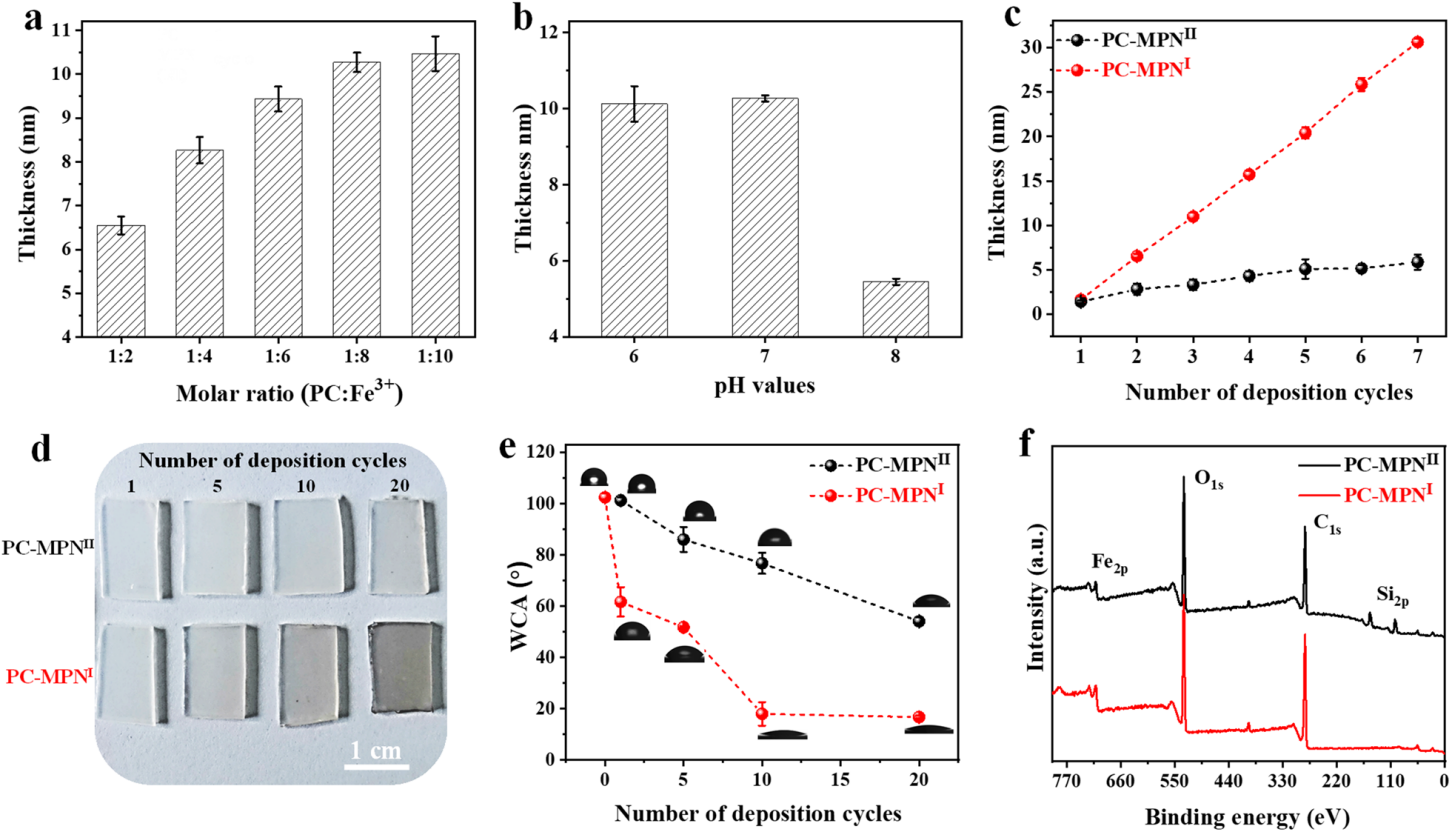
Effect of the **a** molar ratio between PC and Fe^3+^, **b** pH and **c** different engineering routes (route I and route II) on the thickness of the PC-MPN coating on silicon. **d** Photographs and **e** water contact angle of the PC-MPN^I^ and PC-MPN^II^ coatings on PP sheets with different deposition cycles. **f** XPS spectra of PC-MPN^I^ and PC-MPN^II^ coatings on silicon. The numbers of deposition cycles were 3 for **a** and **b** and 7 for **f**

PC-MPNs could be successfully coated onto bare substrates through two routes depending on the priority of PC or Fe: route I, in which PC solution was added first followed by Fe^3+^; and route II, in which Fe^3+^ solution was added first followed by PC. To understand the deposition mechanism of PC-MPNs, factors that affect the growth behavior of PC-MPNs, including the pH, molar ratio between PC and Fe^3+^, and different routes (route I and route II in Scheme 1b), were investigated. To avoid the interplay between these factors, only one factor was varied, while the other factors remained unchanged. As displayed in Fig. 1a, in pH 6.5 buffer, the thickness of the PC-MPN^I^ coating gradually increased within increasing molar ratio ranges from 1:2 to 1:10, and a further increase in Fe^3+^ led to a plateaued thickness. As shown in Fig. 1b, under a fixed molar ratio of 1:10, the thickness of the PC-MPN^I^ coating decreased when the pH value increased from pH 6 to 8, which may be due to the weak interaction between PC and the substrate at high pH, thus making it difficult to form an MPN coating. Using an optimized molar ratio (PC:Fe^3+^, 1:10) and pH 6.5, the engineering routes (route I and route II) for the PC-MPN coating were inspected. We found that the growth of the PC-MPN coating was greatly affected by the engineering route (route I and route II). As shown in Fig. 1c, the PC-MPN^I^ coating showed fast linear growth rate of approximately 31 nm after seven deposition cycles, with approximately 4–5 nm per cycle. In contrast, the PC-MPN^II^ coating displayed very slow growth rate at less than 6 nm over seven deposition cycles, with less than 1 nm in each cycle. At the same numbers of deposition cycles, the PC-MPN^I^ coating displayed approximately 5 times thicker than PC-MPN^II^ coating, as indicated in Fig. 1c.

To further investigate the efficiency of the two assembly coatings, PC-MPNs were coated onto transparent PP sheets. This efficiency can be clearly observed from the color change in Fig. 1d. Compared with the PC-MPN^II^ coating, the color of the PC-MPN^I^ coating deepened more significantly as the number of deposition cycles increased; moreover, route I was much more effective than route II for PC-MPN coating formation. This finding could also be verified from the changes in the WCA, a sensitive technique to surface hydrophilicity. Due to the hydrophilicity of PC and ferric ions, the PC-MPN coating formed onto hydrophobic substrates would reduce its WCA. Taking the substrate of PP sheet as an example (Fig. 1e), the WCA of the PC-MPN^I^ coating was much lower than that of the PC-MPN^II^ coating at the same number of cycles. And the WCA of PC-MPN^I^ coating decreased much faster than that of PC-MPN^II^ coating with the numbers of cycles. The WCA of PC-MPN^I^ coating reached to flat (approximately 18 degrees) using less than 10 deposition cycles, while PC-MPN^II^ coating was far beyond the terrace (approximately 54 degrees even at 20 deposition cycles).

For PC-MPNs coated on a bare silicon wafer by route I and route II, the presence of Fe_2p_ signals indicated the successful deposition of PC-MPNs, as tested by XPS (Fig. 1f), which is a technique offer precise measurements for ultra-thin (< 3 nm) films. The Si_2p_ signal from the underlying substrate was clearly detected in the PC-MPN^II^ coating (Fig. 1f, top) but not in the PC-MPN^I^ coating (Fig. 1f, bottom), suggesting a thicker coating formed by the PC-MPN^I^ coating. All these data (Figs. 1c–f) indicated that route I was much more effective than route II in fabricating PC-MPN coatings. In the following, the chemical composition and the constructive mode of the PC-MPN^I^ and PC-MPN^II^ coatings were investigated by XPS, and more than one hundred cycles were deposited onto silicon to avoid disturbance of the underlying substrate (Additional file 1: Figure S2). As expected, the silicon signal disappeared in both the PC-MPN^I^ and PC-MPN^II^ coatings, suggesting that the XPS signals were originated from the coatings (Additional file 1: Figure S2).

**Fig. 2:**
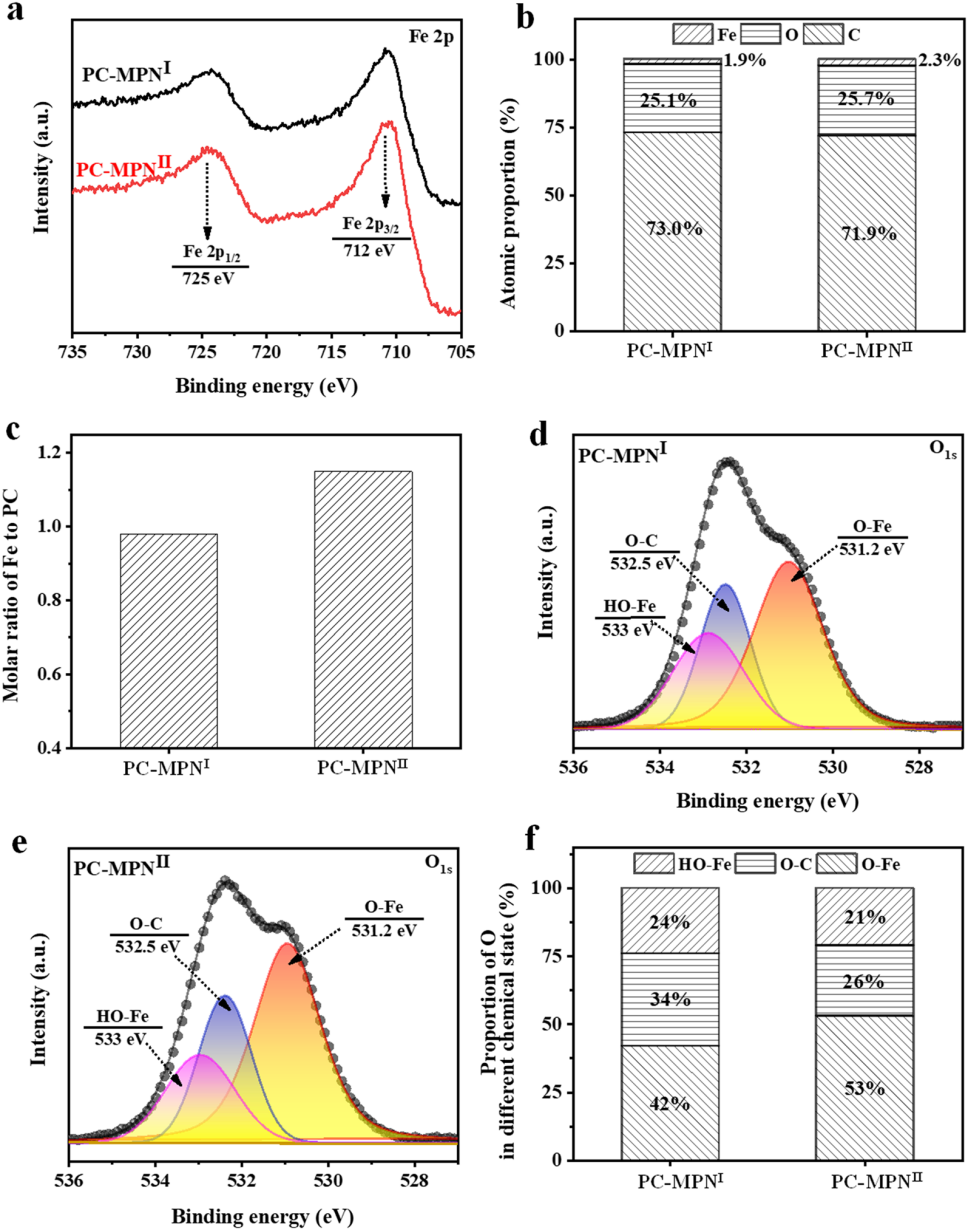
**a** XPS spectrum, **b** atomic proportion, and **c** molar ratio of Fe to PC in the PC-MPN^I^ and PC-MPN^II^ coatings. Detailed O 1 s spectra of the **d** PC-MPN^I^ and **e** PC-MPN^II^ coatings. **f** Proportion of O in different chemical states in the PC-MPN^I^ and PC-MPN^II^ coatings

In the detailed Fe_2p_ spectra (Fig. 2a), two split peaks at 725 eV and 712 eV were observed, indicating the presence of Fe^3+^ in both PC-MPN^I^ and PC-MPN^II^ coatings. The atomic percentages of carbon (C), oxygen (O) and iron (Fe) are illustrated in Fig. 2b, and no big difference between PC-MPN^I^ and PC-MPN^II^ coatings was observed, except for the slight increase in Fe content (1.9% in PC-MPN^I^ coating and 2.3% in PC-MPN^II^ coating). Furthermore, the generated molar ratios of Fe to PC in PC-MPN^I^ and PC-MPN^II^ coatings were 0.98 and 1.15 (Fig. 2c), respectively. In the detailed O 1 s spectra (Fig. 2d of PC-MPN^I^ coating and Fig. 2e of PC-MPN^II^ coating), three peaks were observed: Fe–O [38, 39] (coordination bond between PC and Fe) at 531.2 eV, O-C in PC at 532.5 eV and Fe-OH [22, 40] (hydrogen bonding interaction between PC and Fe) at 533 eV. After the peak fit, the proportion of O in different states was obtained. As shown in Fig. 2f, compared to the PC-MPN^I^ coating, the PC-MPN^II^ coating had a higher proportion of coordinative bonds (53% in the PC-MPN^II^ coating vs. 42% in the PC-MPN^I^ coating) but a lower proportion of O-C bonds (26% in the PC-MPN^II^ coating vs. 34% in the PC-MPN^I^ coating), suggesting more coordinative interactions and less PC in the PC-MPN^II^ coating. Overall, these two coatings had almost the same chemical composition and were constructed by similar modes but different proportions in constructive mode.

**Fig. 3:**
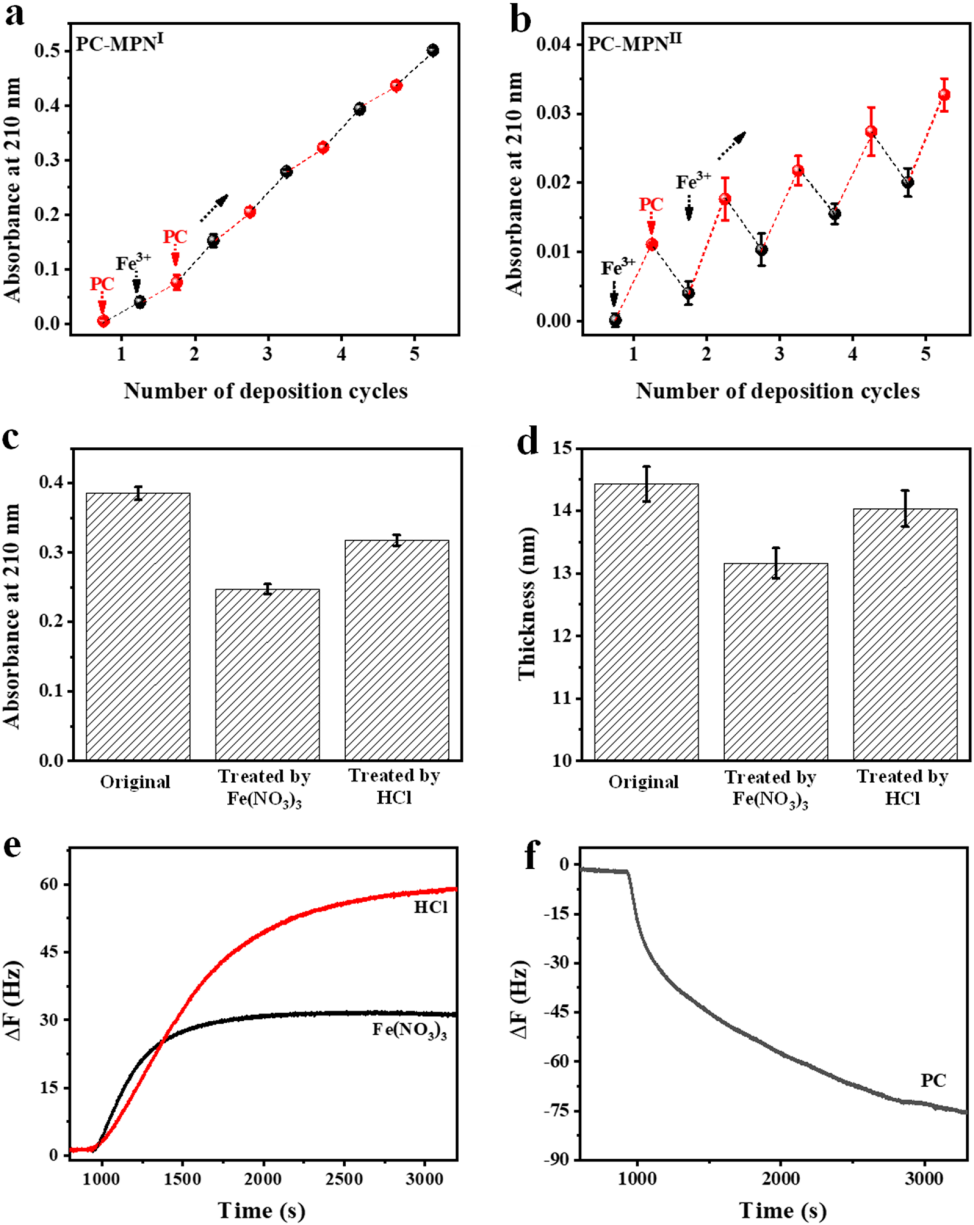
UV absorbance at 210 nm as a function of deposition cycles for **a** PC-MPN^I^ and **b** PC-MPN^II^ coatings. **c** UV absorbance at 210 nm and **d** coating thickness of the PC-MPN^I^ coating before and after treatment with Fe(NO_3_)_3_ and HCl solutions. Frequency shift Δf of the PC-MPN^I^ coating in **e** Fe(NO_3_)_3_ and HCl and **f** PC solutions

To further investigate the reasons for the above differences (coating efficiency and constructive mode) between the PC-MPN^I^ and PC-MPN^II^ coatings, UV–vis was utilized to observe the engineering steps in each cycle, and the absorbance at 210 nm associated with the absorption of phenyl groups in PC was used to quantify the PC in the coating (Additional file 1: Figs. S3 and S4). For the PC-MPN^I^ coating (Fig. 3a), there was a continuous increase at 210 nm every step in each cycle, regardless of whether PC or Fe^3+^ was added. However, the PC-MPN^II^ coating exhibited a zigzag-type increase (Fig. 3b), with an increase in the step of adding PC but a decrease in the step of adding Fe^3+^, which was totally different from the growth profile of the PC-MPN^I^ coating. The absorbance of the PC-MPN^II^ coating was much lower than that of the PC-MPN^I^ coating at the same number of deposition cycles. These data (Figs. 3a, b) suggested that although the PC-MPN coating can be generated by both route I and route II, PC-MPN^I^ was much more efficient than PC-MPN^II^, which was consistent with the above thickness test (Fig. 1c). The low coating efficiency of PC-MPN^II^ was attributed to the decomposition of pre-deposited PC-MPNs during the step of adding Fe^3+^, and only 40% of the newly deposited coating in the last cycle was retained (Additional file 1: Figure S5, deduced from the data in Fig. 3b). Next, the decomposition process of preformed PC-MPNs induced by Fe^3+^ was explored to deeply understand the formation mechanism of the PC-MPN coating. Considering that the MPNs are extremely sensitive to acidic environments [41] and that the Fe(NO_3_)_3_ solution used here was pH 2.6, a PC-MPN^I^ coating with a thickness of approximately 14 nm was separately immersed into dilute HCl at pH 2.6 and Fe(NO_3_)_3_ solutions for 5 min to separately reveal the roles of acidic environments and Fe^3+^ in this decomposition process. UV–vis was recorded before and after immersion (Additional file 1: Figure S6). Decreased absorbance was found after both treatments, and the Fe(NO_3_)_3_ treatment resulted in a greater decrease than the dilute HCl treatment (Fig. 3c). The thickness change in the PC-MPN^I^ coating by the same treatment could support UV–vis data. As exhibited in Fig. 3d, treatment with Fe(NO_3_)_3_ led to a greater decrease in thickness than treatment with dilute HCl. The average decomposition rate could be calculated based on the data in Figs. 3c, d. The decomposition rate induced by Fe(NO_3_)_3_ was approximately 2.2 times faster than that induced by dilute HCl based on the UV–vis data in Fig. 3c, and it was approximately 2.4 times faster based on the thickness data in Fig. 3d. Besides the acidic environment in Fe(NO_3_)_3_ solution, excessive Fe^3+^ competing with the PC in the preformed coating attributed to the decomposition, which resulted in faster destruction of PC-MPNs. These data strongly showed that both an acidic environment and Fe^3+^ played a role in breaking the pre-deposited PC-MPN coating.

Then, QCM-D, a real-time observation technique with sensitivity to a picometer of the coating on its sensor, was performed to investigate this decomposition process. After injecting dilute HCl or Fe(NO_3_)_3_ into the chamber in which the PC-MPN^I^ coating was coated onto the sensor, the frequency significantly increased with time (Fig. 3e), suggesting the decomposition of the PC-MPN coating. Compared to dilute HCl, Fe(NO_3_)_3_ showed a faster decomposition rate, especially in the first 5 min, which was consistent with the UV–vis observations. In addition, Fe(NO_3_)_3_ experienced a quicker equilibrium process with less decomposed material than dilute HCl. In contrast, the injection of PC resulted in a continuously decreased frequency (Fig. 3f), suggesting the deposition of PC onto the PC-MPN coating. Obviously, during engineering of the PC-MPN^I^ and PC-MPN^II^ coatings, the total performance of Fe(NO_3_)_3_ and PC on the PC-MPN coating leads to different coating efficiencies and constructive modes of the MPN coating.

**Fig. 4:**
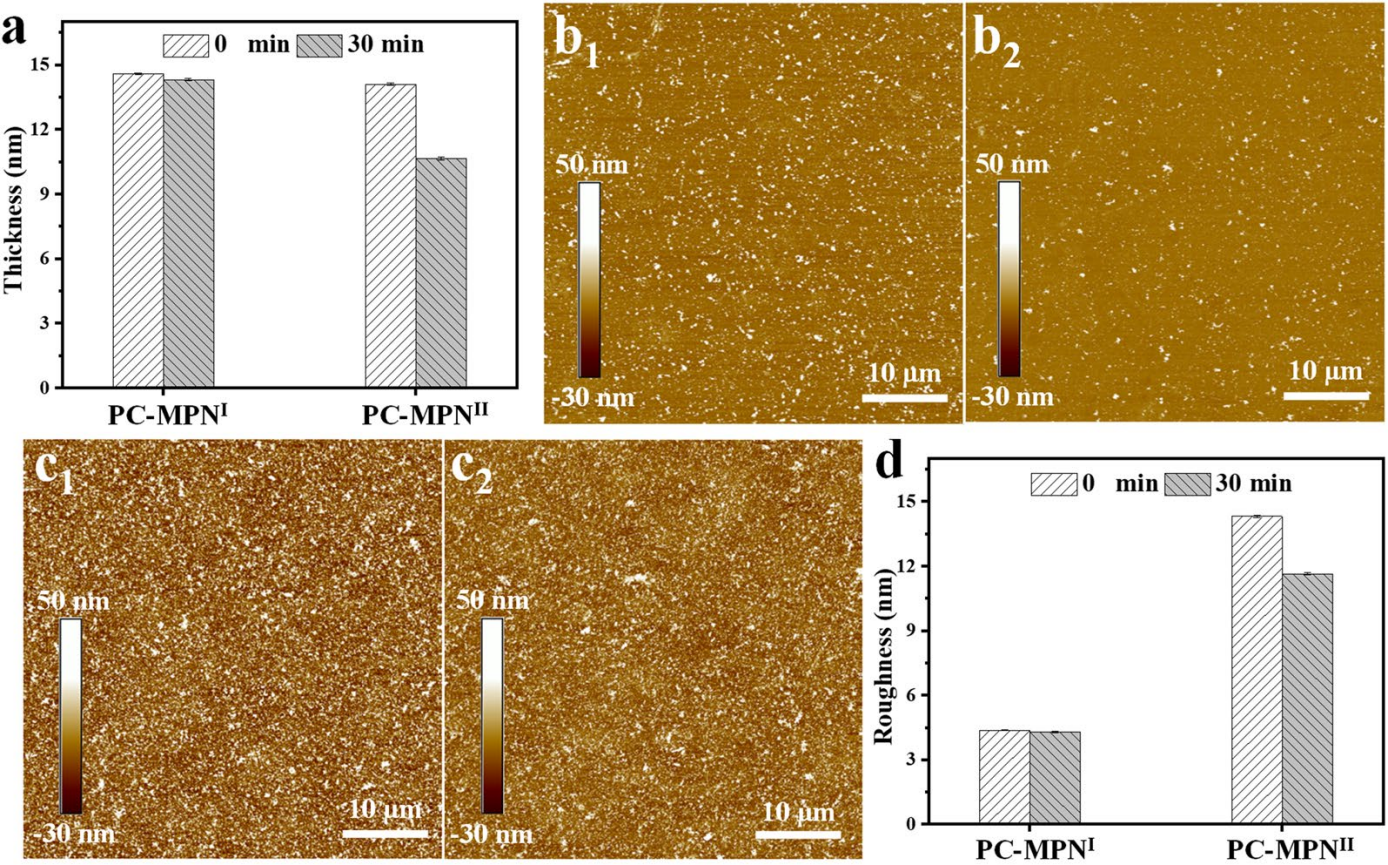
**a** Coating thickness of the PC-MPN^I^ and PC-MPN^II^ coatings before and after treatment with Fe(NO_3_)_3_ for 30 min. AFM images of **b** PC-MPN^I^ and **c** PC-MPN^II^ coatings before and after treatment with Fe(NO_3_)_3_ for 30 min. b_1_, c_1_ and b_2_, c_2_ are images taken before and after treatment, respectively. **d** Surface roughness of the PC-MPN^I^ and PC-MPN^II^ coatings before and after treatment with Fe(NO_3_)_3_ for 30 min

The stability of the PC-MPN^I^ and PC-MPN^II^ coatings was further checked by immersing them into Fe(NO_3_)_3_ solution for 30 min. As displayed in Fig. 4a, from the original similar thickness (~ 14 nm), the thickness of both PC-MPN^I^ and PC-MPN^II^ coatings decreased. Compared to the PC-MPN^II^ coating, the PC-MPN^I^ coating showed a much smaller decrease, which was further proved by the percent of reserved thickness (Additional file 1: Figure S7). More than 98% of the coating was reserved for PC-MPN^I^, while only 75% was left for PC-MPN^II^. The morphologies of these coatings before and after immersion in Fe(NO_3_)_3_ solution for 30 min were recorded by AFM, which is a technique used to study the film in the 1–20 nm thickness range. As shown in Figs. 4b, c, all coatings were uniform and presented a particle-like height structure on the surface, both before and after immersion. The morphology of the PC-MPN^I^ coating seemed flatter than that of the PC-MPN^II^ coating (Fig. 4b vs. Fig. 4c), and the height structure of the PC-MPN^II^ coating decreased obviously after immersion (Fig. 4c1 vs. Fig. 4c2). The roughness of the PC-MPN^I^ coating had no obvious change before and after immersion (Fig. 4d). There was a significant decrease in the PC-MPN^II^ coating after immersion (Fig. 4d). All these data (Figs. 4a–d) suggested that the PC-MPN^I^ coating was more stable than the PC-MPN^II^ coating in resisting the erosion of Fe(NO_3_)_3_.

**Scheme 2:**
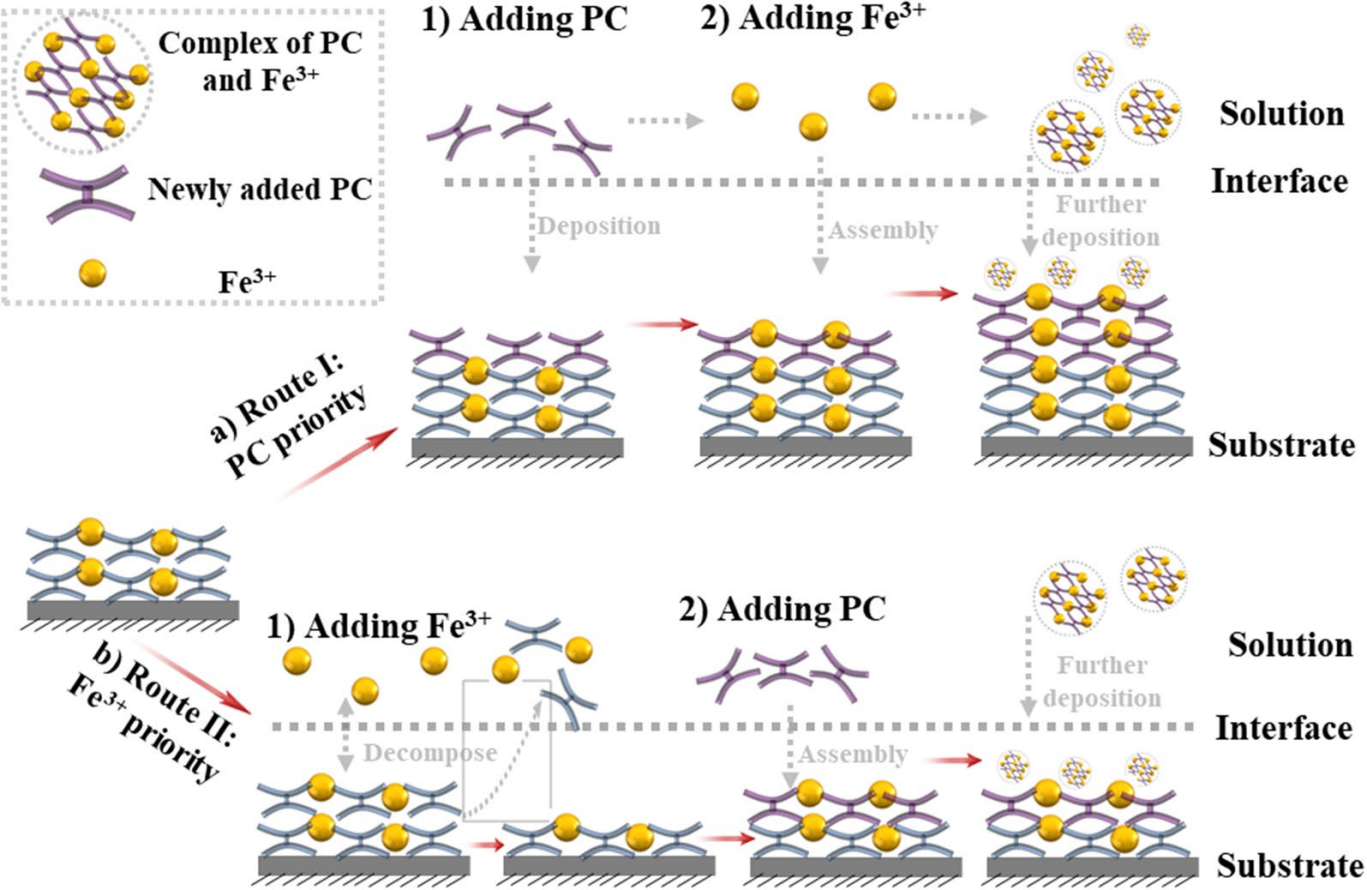
Proposed mechanism for the formation of the **a** PC-MPN^I^ and **b** PC-MPN^II^ coatings

Based on the above results, the formation mechanisms of PC-MPN^I^ and PC-MPN^II^ coatings were proposed. As illustrated in Scheme 2a, adding PC first led to PC deposition onto the preformed PC-MPN coating and increased the thickness, and the subsequent addition of Fe^3+^ contributed to a further thickness increase by assembly with the pre-deposited PC; meanwhile, the complex of PC and Fe^3+^ in solution was further deposited onto the PC-MPN coating. According to the generation of the PC-MPN^II^ coating in Scheme 2b, adding Fe^3+^ first resulted in the decomposition of the preformed PC-MPN coating, and adding PC second led to the deposition of PC onto the substrate and the formation of the complex of PC and Fe^3+^ in solution, which could be further deposited onto the substrate. The cause of the decomposition of the PC-MPN coating by Fe^3+^ priority was that the dynamic coordinative balance between PC and Fe^3+^ in the coating was broken by the low pH and presence of Fe^3+^. Compared with the Fe^3+^ priority, the PC priority did not result in decomposition of the preformed PC-MPN coating, but the deposition might be the hydrophobic interaction and hydrogen bonding between PC in PC-MPNs and PC in solution. Further deposition of the PC and Fe^3+^ complex onto the substrate could be supported by the SEM images and roughness of the coating (Figs. S8, S9). As exhibited in Additional file 1: Figure S8, the number of particles on the surface of both PC-MPN^I^ and PC-MPN^II^ coatings obviously increased with increasing number of deposition cycles. In Additional file 1: Figure S9, the roughness tested by AFM increased with increasing number of deposition cycles. It is worth noting that the PC-MPN^I^ coating had a higher roughness than the PC-MPN^II^ coating with the same number of deposition cycles.

It is worth noting that the phenomena of the material priority-dominated PC-MPN coating were opposite to those of the most studied TA-MPN coating, in which the priority of Fe^3+^ resulted in an efficient process while the priority of polyphenol TA was an inefficient coating route [20, 21]. This might be mainly attributed to the difference in backbone between phenolic acids (TA, C6-C1) and flavonoids (PC, C6-C3-C6) and the different applied pH values during MPN formation.

**Fig. 5:**
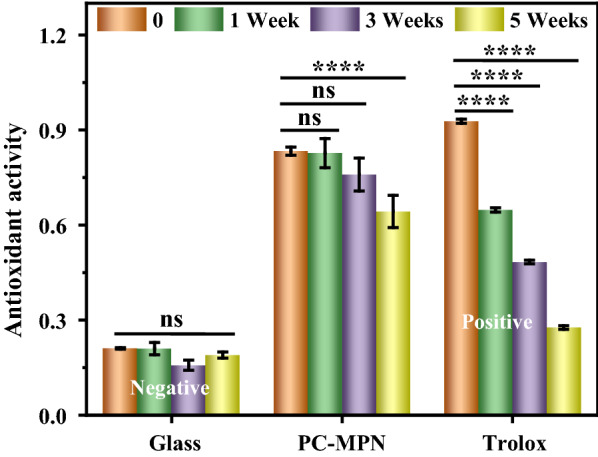
Antioxidant activity of glass and PC-MPN coating as a function of stored time

Because of the multifunctionality presented by PC (antioxidant, antibacterial, insect pest resistance, antiobesity, etc.), the performance of PC-MPNs as a bioactive interface platform was investigated. In the following studies, only PC-MPN^I^ coating was utilized to coat the substrate due to its high coating efficiency and relative stability. The antioxidant activity of the PC-MPN coating was first evaluated by the FRAP assay. Based on the tested intensity, the antioxidant activity of each sample was calibrated from the standard curve (Additional file 1: Figure S1). Glass and trolox were run as the negative and positive control groups. The antioxidant activities were revealed in the short term and the long term by testing the antioxidant activities of the PC-MPN coating at different time intervals (i.e., freshly prepared and stored for 1, 3, and 5 weeks in water). As shown in Fig. 5, the PC-MPN coating is comparable to the trolox and exhibit much higher antioxidant activity than the glass control, which demonstrated that the coating was responsible for the antioxidant activity of the samples. Notably, the antioxidant activity of trolox decreased rapidly with the increasing of stored time, while there was no obvious difference for PC-MPN coating within 3 weeks, which is crucial for future clinical applications.

**Fig. 6:**
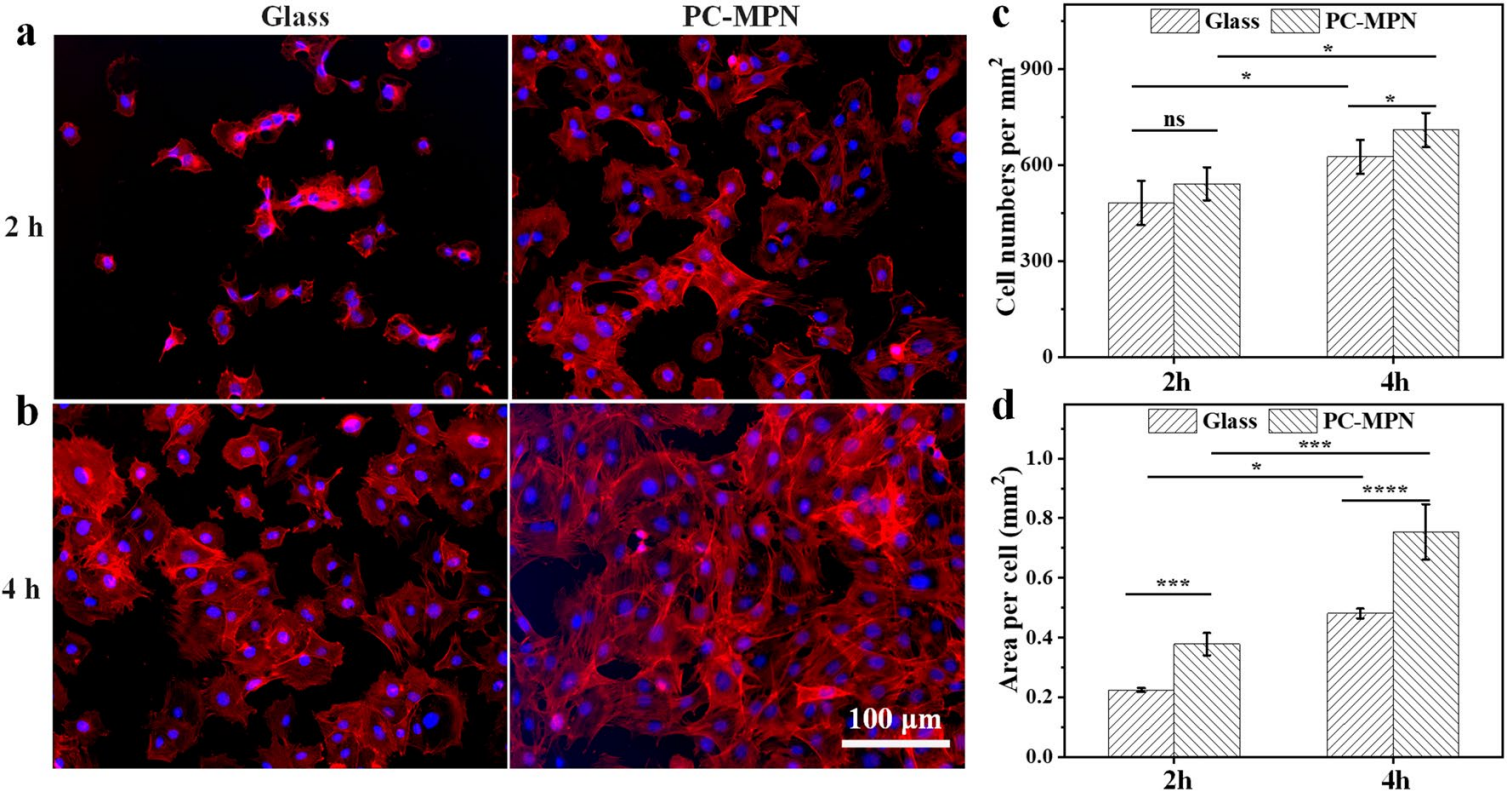
Adhesion of cells to the PC-MPN coating: **a**, **b** Fluorescence microscopy images of MC3T3-E1 cells seeded on glass and PC-MPN coating for 2 and 4 h. **c** Cell number per square millimeter and **d** area per cell of the seeded MC3T3-E1 on glass and PC-MPN coating at 2 h and 4 h

The early and long-term adhesion of cells on the surface of materials is an important index for evaluating the effect of the interaction between materials and cells. Quick cell attachment and spreading onto implants at an early stage are beneficial for integrating tissue cells and mitigating the effect of bacteria [42–45]. Cell attachment onto the PC-MPN coating at an early stage was observed by observing the behavior of MC3T3-E1 cells at 2 h and 4 h after seeding by fluorescent images, and the cells seeded on glass were used as the control. As shown in Figs. 6a, b, the cells on the surface of the PC-MPN coating exhibited slight dispersal and showed a well-defined morphology at both 2 h and 4 h, while the cells on glass were round at 2 h and slightly spread at 4 h.

In a further analysis, the cell number and cell area per square millimeter, which are two of the most important indices for evaluating attachment at early stage, were also quantified, as plotted in Figs. 6c, d. Obviously, compared with that at 2 h, both the cell number and cell area per square millimeter increased at 4 h in all samples. Significantly, more cells adhered to the PC-MPN coating than glass at both 2 h and 4 h. The cell numbers on the coating were all 1.3-fold higher than those on glass at both 2 h and 4 h (Fig. 6c). For the area per square millimeter (Fig. 6d), cells on the PC-MPN coating also had a higher area than that on glass, and the values were 1.78-fold and 1.44-fold higher than that on glass at both 2 h and 4 h, respectively. All the above results showed that the PC-MPN coating provided a microenvironment for the rapid attachment and dispersal of MC3T3-E1 cells at an early stage.

**Fig. 7:**
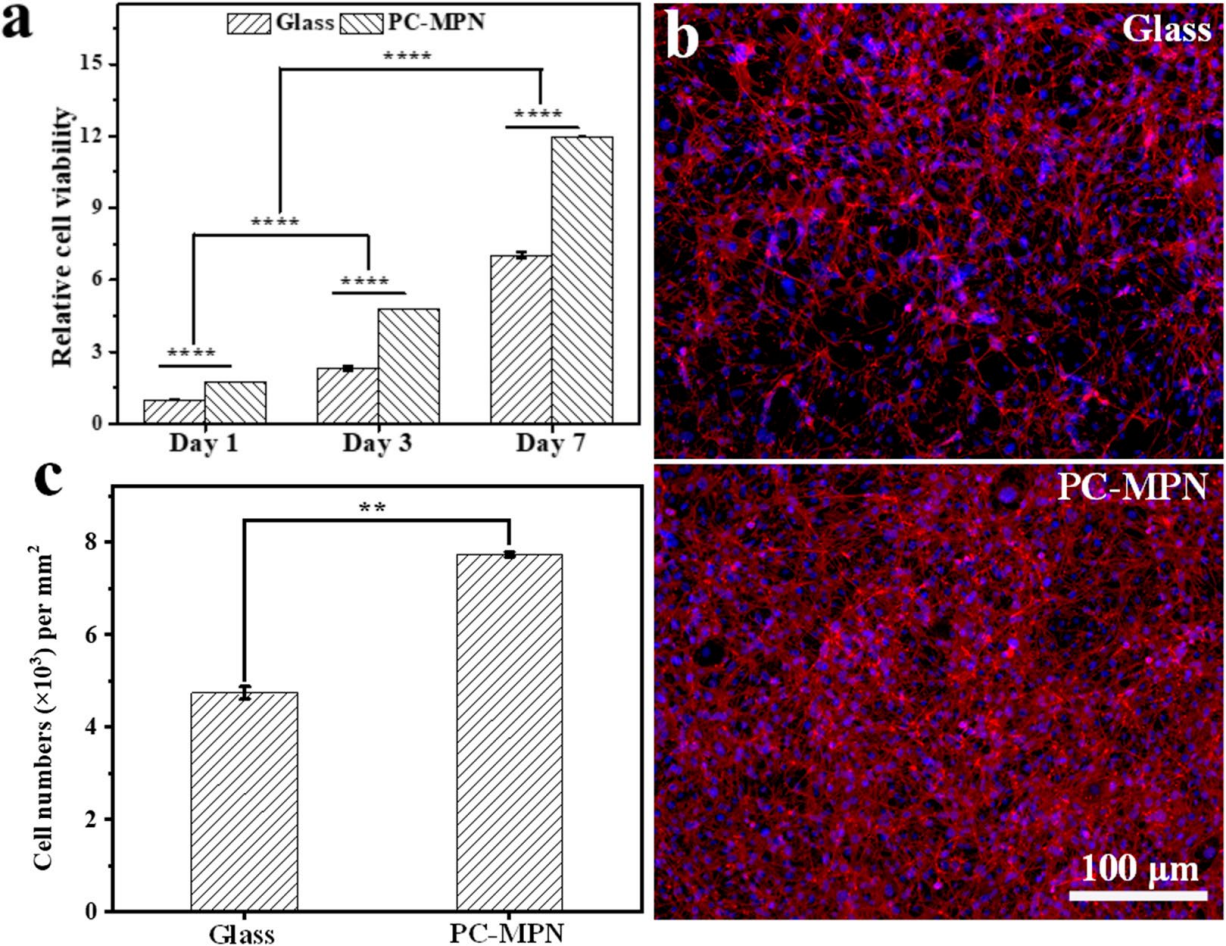
Proliferation of cells on the PC-MPN coating: **a** Relative cell activity of MC3T3-E1 cells seeded on glass and the PC-MPN coatings for 1, 3 and 7 days. **b** Fluorescence microscopy images of MC3T3-E1 cells seeded on glass and the PC-MPN coating. **c** Cell numbers per square millimeter onto glass and the PC-MPN coating for 7 days

To investigate the proliferation of MC3T3-E1 cells on PC-MPN coatings, a longer culture time was carried out, and cell viability (an indicator of cell numbers) was tested on days 1, 3, and 7. The number of proliferated cells was evaluated from the OD values of the CCK-8 assay, which was based on dehydrogenase activity detection in viable cells to count the number of cells. The OD value of the CCK-8 assay was proportional to the number of viable cells. With the cell viability on glass at day 1 as the control, the cellular viabilities are shown in Fig. 7a. Obviously, the cell viabilities increased with culture time in all samples, suggesting that all seeded cells settled on all samples in a similar way and began proliferating normally. At any culturing time, the number of cells on the coating was higher than that on glass, demonstrating the good proliferation of cells on the PC-MPN coating. On the 7th day, the cells were stained and imaged (Fig. 7b). The cell numbers were counted based on the number of cellular nuclei in Fig. 7b and are displayed in Fig. 7c. Clearly, we observed that a higher number of cells adhered to the PC-MPN coating than glass after 7 days of culture, which was consistent with the CCK-8 assay (Fig. 7a). In detail, the cell viability on the PC-MPN coating was approximately 1.7 times higher than that on glass at the same time point. Considering the infinite cell numbers under in vivo conditions, these features of fast cell attachment and spreading at an early stage and proliferation in the initial days indicate that the PC-MPN coating is a good candidate for attracting more cells to attach, spread and proliferate.

**Fig. 8:**
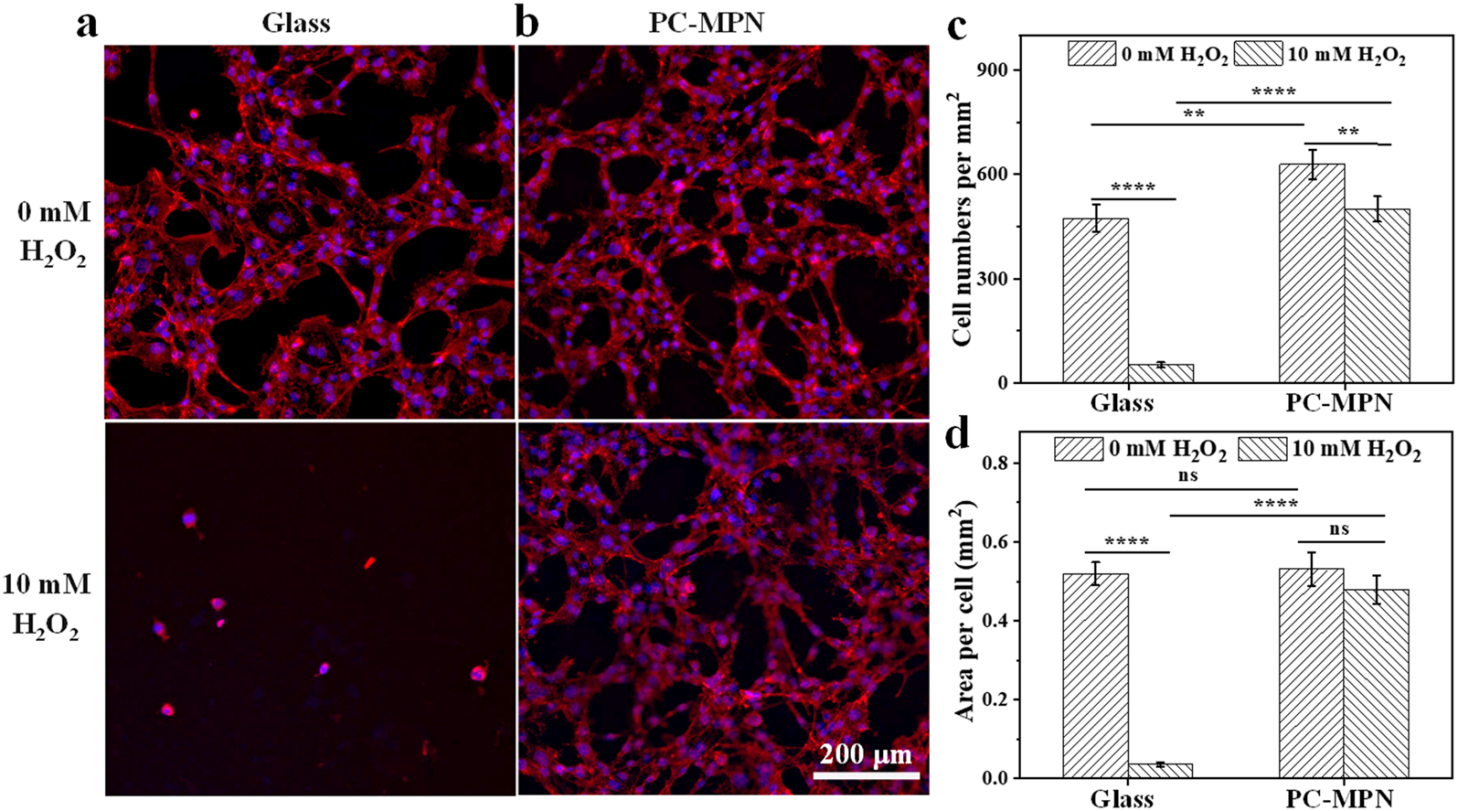
Antioxidant profile of the cell response: **a**, **b** Fluorescence microscopy images of MC3T3-E1 cells seeded on glass and PC-MPN coating. **c** Cell numbers per square millimeter and **d** area per cell on glass and PC-MPN coating before and after 10 mM H_2_O_2_ treatment

Polyphenols have good antioxidant ability through multiple mechanisms, such as scavenging free radicals [46], activating detoxifying/defensive proteins [47], etc. PC has been reported as an activator to antioxidative enzymes and the glutathione cycle [48], especially after chelation with metal ions under suitable conditions [30, 49], therefore, a PC-MPN coating was chosen to further investigate the antioxidant properties of the cell response. To simulate an overstressed ROS environment [50, 51], 10 mM H_2_O_2_ was added to the culture medium as an ROS provider. As displayed in the top panel of Figs. 8a, b, the cells had extensively spread onto all samples before H_2_O_2_ treatment. After treatment with H_2_O_2_ for 20 min (bottom panel of Figs. 8a, b), the cells on glass became skinny, and the number of attached cells decreased accordingly. Interestingly, although the cell area and number of cells that adhered to the coating also changed after H_2_O_2_ treatment, the trend of change was slighter than that of the glass. Further analysis of the difference was quantified by counting the number of cells and measuring the cell area per square millimeter. As displayed in Fig. 8c, the cell number per square millimeter on glass exhibited a sudden decrease, with more than 89% of the original attached cells lost after H_2_O_2_ treatment. As a comparison, more than 80% of the original attached cells remained attached to the coating. The cell area per square millimeter is plotted in Fig. 8d, and a decreased area was observed for all samples. However, the area of decrease was 93% and 10% for glass and the coating, respectively. Evidently, cells attached to the PC-MPN coating had a stronger ability to resist oxidative stress than those on glass.

**Fig. 9:**
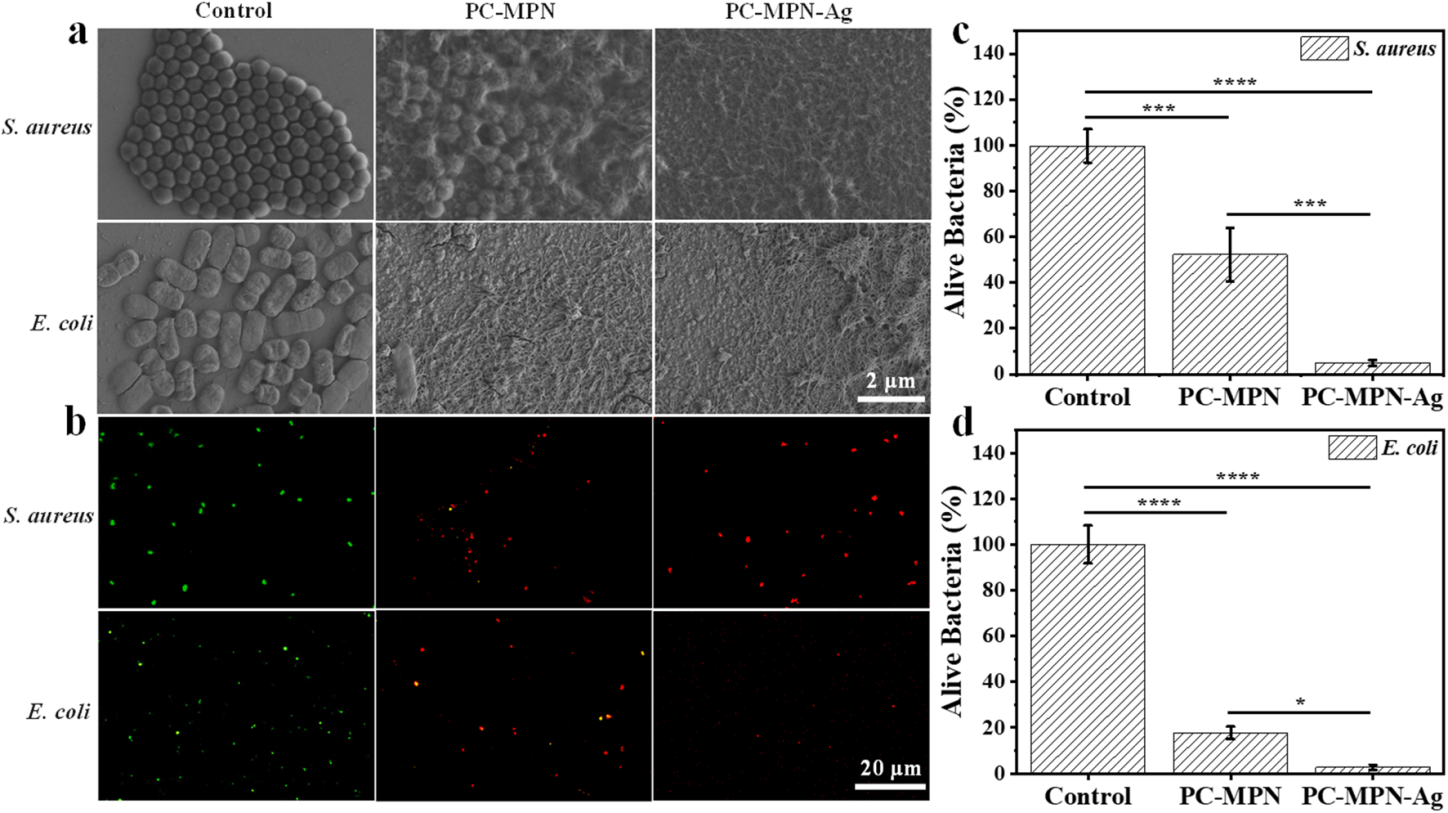
Antibacterial properties of the coating: **a** SEM and **b** fluorescence microscopy images of *S. aureus* and *E. coli* on substrates, PC-MPN coating and PC-MPN-Ag coating. **c**, **d** The percentage of alive *S. aureus* and *E. coli* counted from the fluorescence micrographs

Bacterial infections, especially in connection with the use of medical diagnostic devices and therapeutic tools/implants, are among the main causes of death worldwide [10]. To address the issue of infections, effective antibacterial materials are still highly sought. Since both polyphenols [31, 52–54] and iron [55, 56] have the ability to kill bacteria, we next tested the antibacterial properties of the coating. The ability of the PC-MPN coating to kill bacteria was tested by immersing the coating into bacterial solutions and then comparing the deformed and collapsed bacteria to those on the silicon wafer control. To show the power of the coating to kill bacteria, two bacteria were chosen as representatives: *S. aureus*, which represents gram-positive bacteria, and *E. coli*, which represents gram-negative bacteria. We employed SEM to characterize the antibacterial activity of the PC-MPN coating. As vividly displayed in Fig. 9a, on the control silicon wafer, cells of *S. aureus* appeared intact, had a round shape and tended to form colonies with each other. No visible abnormalities were observed. However, bacteria in contact with the PC-MPN coating showed deformation, collapsed bacterial structures and fusion of the bacterial membrane; in addition, islands with ruptured bacteria occurred, thus revealing severe damage to the bacterial structure. The mechanism for antibacterial property of PC-MPN may be attributed to that PC can bind to bacterial membrane, inhibit the synthesis of peptidoglycan and damage the bacterial morphology in following. Cells of *E. coli* appeared to have similar results to those for *S. aureus*. Notably, Ag^+^ is well known for its multilevel antimicrobial mode, even at very low concentration levels, which ensures wide-spectrum and long-term antibacterial characteristics. Taking the reducing ability of residual phenolic hydroxyl groups on the PC-MPN coating, silver ions can be reduced to silver nanoparticles in situ with good antibacterial activity. Furthermore, we also explored the antibacterial properties of the PC-MPN-Ag coating. Distinctly, both *S. aureus* and *E. coli* displayed further fusion of the bacterial membrane on the PC-MPN-Ag coating, indicating that the PC-MPN-Ag coating showed stronger bactericidal ability (Fig. 9a).

To further confirm the antibacterial activity, the SYTO 9/PI method was implemented, with dead bacteria dyed red and live bacteria dyed green. As displayed in Fig. 9b, bacteria attached to the PC-MPN and PC-MPN-Ag coatings were red, while bacteria attached to the control (glass) were green. Notably, compared with glass, the number of dead and live bacteria on the PC-MPN-Ag coating showed a decreasing tendency, especially for *E. coli*, which may be due to the strong antibacterial property of the PC-MPN-Ag coating preventing the bacteria from firmly adhering to the coating surface and reducing proliferation. In a further analysis, the percentage of alive bacteria was also evaluated, as shown in Figs. 9c, d. Clearly, the percentage of both alive *S. aureus* and *E. coli* all decreased on PC-MPN coating and further decreased on PC-MPN-Ag coating. All of these results further illustrated that the PC-MPN coating, particularly the PC-MPN-Ag coating, has strong antibacterial ability against *S. aureus* and *E. coli*.

Nanometer-thick coating (nanocoating) is multifunctional in various fields, making it possible to control surface properties including controlled roughness, stiffness and bioactivities [57, 58]. Nanocoating can ensure film uniformity for nano/micrometer-sized objects. The material-independent, universal MPN nanocoating have been extensively used in biomedical materials, environmental science, and daily life, such as antibacterial coating of biomedical devices [59], anti-moisture coating of glasses [60], water–oil biphasic separation [19], inorganic salt nanofiltration membrane [61] and facile inking for writing [62], etc. Although there are various fascinating functionalities endowed by MPN nanocoatings, they are largely confined to phenolic acid TA-MPN system and usually one-sided functionality. However, many applications, especially for implants, require dual or even multiple functionalities, such as biocompatible and antibacterial materials. Biocompatible surfaces enhance cell adhesion, proliferation, and tissue integration [63], while the antibacterial surface greatly reduces the incidence rate of peri-implantitis induced by chronic bacterial infection [64]. Considering the property of MPNs is mainly determined by the polyphenols used, herein, we constructed a flavanol-based PC-MPN system and systemically investigated their bioactivities. PC-MPN coatings can endow the surface with both biocompatibility and antibacterial activity, which can be used as a promising candidate for implant materials. Interestingly, PC-MPN system here can also be employed as a secondary reaction platform by in suit reducing silver ions to sliver nanoparticles taking reducing ability of residual phenolic hydroxyl groups on PC-MPNs and the PC-MPN-Ag coating showed stronger bactericidal ability than PC-MPNs. Our work greatly broadens the MPN systems and can be useful for a range of applications including biosensing, drug delivery and tissue engineering especially for functional nanocoating of implants.

## Conclusions

In summary, we successfully engineered flavonoid-based multifunctional MPNs constructed from PC and Fe^3+^. The desired PC-MPN coating was obtained by optimizing assembly parameters, such as pH, polyphenol-to-metal molar ratio and polyphenol-to-metal priority in each deposition cycle. For the PC priority (PC-MPN^I^), the thickness was 5 times higher than that of the Fe^3+^ priority (PC-MPN^II^). The attachment of PC onto the substrate or preformed PC-MPN coating was critical to the continuous and accelerated growth of the PC-MPN^I^ coating, while the decomposition of dilute HCl and Fe^3+^ led to a zigzag pattern and slower growth of the PC-MPN^II^ coating. In addition to the high coating efficiency, the PC-MPN^I^ coating exhibited a higher stability in terms of resistance to Fe^3+^ erosion than the PC-MPN^II^ coating. Furthermore, the PC-MPN coating greatly promoted cell adhesion and proliferation and antioxidative and antibacterial activities. We believe that these findings could reveal the formation mechanism of MPNs and be helpful for the future design of MPN-related coatings, and the strategy of engineering PC-MPN coatings with multifunctionalities could pave the way to considerable applications, such as in sensors, the environment, drug delivery, and tissue engineering.

## Supplementary Information

**Additional file 1: Figure S1.** Standard curve of antioxidant activity.** Figure S2.** XPS spectrum of PC-MPN^I^ and PC-MPN^II^ coatings with 100 deposition cycles. **Figure S3.** The growth of PC-MPN^I^ coating. **Figure S4.** The growth of PC-MPN^II^ coating. **Figure S5.** The decomposition of the PC-MPN^II^ coating: ${\text{A}}_{{Fe}^{3+}}$ and A_PC_ are the decreased absorbance and increased absorbance in the same cycles, respectively. **Figure S6.** UV-vis spectrum of PC-MPN^I^ coating before and after immersing in HCl and Fe(NO_3_)_3_ solutions. **Figure S7.** The percent of reserved thickness of PC-MPN^I^ and PC-MPN^II^ coatings after immersing in in Fe(NO3)3 solution for 30 min. **Figure S8.** SEM images of the PC-MPN^I^ and PC-MPN^II^ coatings with different deposition cycles. **Figure S9.** Roughness of PC-MPN^I^ and PC-MPN^II^ coatings.

## Data Availability

The datasets used and analyzed during the current study are available from the corresponding author on reasonable request.
