# Supplementary material for: Material priority engineered metal-polyphenol networks: mechanism and platform for multifunctionalities
Source: J Nanobiotechnology. 2022 Jun 3;20:255. doi: 10.1186/s12951-022-01438-1 (PMC9164710; doi:10.1186/s12951-022-01438-1)
Supplement: Supplementary file 1 — Additional file 1: Figure S1. Standard curve of antioxidant activity. Figure S2. XPS spectrum of PC-MPNI and PC-MPNII coatings with 100 deposition cycles. Figure S3. The growth of PC-MPNI coating. Figure S4. The growth of PC-MPNII coating. Figure S5. The decomposition of the PC-MPNII coating: ${\text{A}}_{{Fe}^{3+}}$AFe3+ and APC are the decreased absorbance and increased absorbance in the same cycles, respectively. Figure S6. UV-vis spectrum of PC-MPNI coating before and after immersing in HCl and Fe(NO3)3 solutions. Figure S7. The percent of reserved thickness of PC-MPNI and PC-MPNII coatings after immersing in in Fe(NO3)3 solution for 30 min. Figure S8. SEM images of the PC-MPNI and PC-MPNII coatings with different deposition cycles. Figure S9. Roughness of PC-MPNI and PC-MPNII coatings. [file 12951_2022_1438_MOESM1_ESM.docx]

*Additional file*

**Material Priority Engineered Metal-Polyphenol Networks: Mechanism and Platform for Multifunctionalities**

*Xinxiu Cheng**,**^¶, §, 1^ Yaxin Zhu**,^§, 1^ Sicheng Tang,^†^ Ruofei Lu,^§^ Xiaoqiang Zhang,^§^ Na Li^† *^ and Xingjie Zan^¶, §, †*^*

*^§^*Xinjiang Technical Institute of Physics and Chemistry, Chinese Academy of Sciences, Urumqi 830011, P.R. China.

*^¶^*School of Ophthalmology and Optometry, Eye Hospital, School of Biomedical Engineering, Wenzhou Medical University 270 Xueyuan Road, Wenzhou 325035, P.R. China.

^†^Oujiang Laboratory, Wenzhou Institute, University of Chinese Academy of Sciences, Wenzhou 325001, China.

*^1^*Equally contributing authors.

^*^Corresponding authors. E-mail addresses: X. Zan, [xjzan2000@hotmail.com](mailto:xjzan2000@hotmail.com); N. Li, [lina0701@ucas.ac.cn](mailto:lina0701@ucas.ac.cn).

**Fig. S1** The standard curve of antioxidant activity.


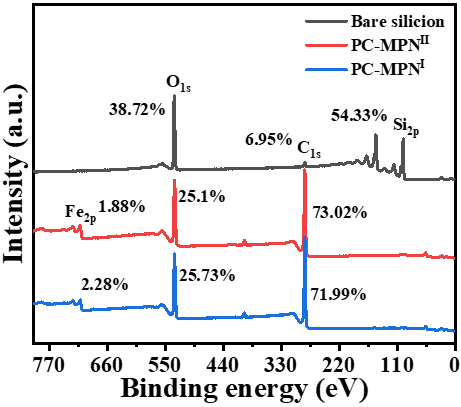


**Fig. S2** XPS spectrum of PC-MPN^Ⅰ^ and PC-MPN^Ⅱ^ coatings with 100 deposition cycles.

**Fig. S3** The growth of PC-MPN^Ⅰ^ coating.

**Fig. S4** The growth of PC-MPN^Ⅱ^ coating.

**Fig. S5** The decomposition of the PC-MPN^Ⅱ^ coating: A_Fe_^3+^ and A_PC_ are the decreased absorbance and increased absorbance in the same cycles, respectively.

**Fig. S6** UV-vis spectrum of PC-MPN^Ⅰ^ coating before and after immersing in to HCl and Fe(NO_3_)_3_ solutions.

**Fig. S7** The percent of reserved thickness of PC-MPN^Ⅰ^ and PC-MPN^Ⅱ^ coatings treated by immersing into in Fe(NO_3_)_3_ solution for 30 min.

**Fig. S8** SEM images of the PC-MPN^Ⅰ^ and PC-MPN^Ⅱ^ coatings in different deposition cycles.

**Fig. S9** Roughness of PC-MPN^Ⅰ^ and PC-MPN^Ⅱ^ coatings.
